# Structural assessment of family and educational influences on student health behaviours: Insights from a public health perspective

**DOI:** 10.1371/journal.pone.0333086

**Published:** 2025-09-25

**Authors:** Zhuo Su, Daifeng Yang, Chun Wang, Zhenlin Xiao, Shanshan Cai

**Affiliations:** 1 School of Transportation, Changsha University of Science and Technology, Hunan, China; 2 School of Computing, Engineering and Digital Technologies, Teesside University, Middlesbrough, United Kingdom; 3 LaFetra College of Education, University of La Verne, La Verne, California, United States of America; 4 Division of Biomedical and Life Sciences, Faculty of Health and Medicine, Lancaster University, Lancaster, United Kingdom; University of Economics Ho Chi Minh City, VIET NAM

## Abstract

The 2024 World Health Organization report reveals that 81% of adolescents worldwide fail to meet the recommended level of physical activity, highlighting a serious global public health challenge. This study approaches the issue from a public health perspective by focusing on the structural determinants of health behavior among university students. Although student health behaviors are shaped by both individual and structural factors, existing research has predominantly emphasized individual-level influences, overlooking the broader educational and systemic context. Employing a structural analysis framework, this study mapped the causal and hierarchical relationships among factors influencing student health behaviors. Data were collected through structured questionnaires administered to undergraduate and postgraduate students in Hunan Province, China. The model identifies family support as the foundational layer in the hierarchy, exerting significant influence on psychological well-being and health motivation, which in turn regulate physical activity and dietary choices. The findings underscore the critical mediating role of educational environments in amplifying the effects of family structures. Based on these insights, this study advocates for the integration of family resources into school-based health promotion interventions, such as digital platforms for parent–student communication, joint family–university health workshops, and collaborative educational health campaigns. Embedding family-oriented strategies into formal education systems may enable universities to enhance student physical and mental well-being in a more integrated and sustainable manner within a public health framework.

## 1. Introduction

Health behaviors such as regular physical activity and balanced nutrition are essential for maintaining the long-term physical and mental well-being of college students. These behaviors significantly reduce the risk of chronic diseases and enhance cognitive function and emotional stability [1]. However, student health behaviors have become an escalating global public health concern. According to the 2024 WHO report, 81% of adolescents worldwide fail to meet recommended physical activity levels. In China, 63% of college students sit for more than six hours a day, only 29% follow dietary guidelines, and 34% report moderate to severe psychological distress [2]. These interrelated issues contribute to a vicious cycle of physical inactivity, poor nutrition, and mental health challenges [3]. More critically, such behaviors are shaped by structural inequalities and environmental constraints. Students from lower socioeconomic backgrounds are 2.3 times more likely to engage in unhealthy behaviors compared to their wealthier peers [4], and limited access to nutritious food and exercise facilities further restricts healthy choices [5]. As these structural factors accumulate, they pose growing risks of early-onset chronic diseases and place increasing pressure on campus health systems [6].

Given the multidimensional and structural nature of health challenges, there is an urgent need to reassess existing research frameworks and analytical methods. In the university context, significant gaps exist in studying student health behaviours, particularly in research frameworks and intervention strategies. Most existing studies and interventions focus too heavily on improving single health behaviours, such as physical activity, dietary habits, or mental health issues, while overlooking the complex interactions between these behaviours. Treating these behaviours as independent variables without considering their interrelationships leads to fragmented intervention outcomes, making it difficult to scale or sustain these interventions in a broader context [7]. Moreover, existing research often neglects the institutional and environmental factors within the university context, which are crucial in shaping student health behaviours. Relying solely on individual-level interventions is insufficient to address these structural issues effectively [8,9].

Traditional analytical models, such as linear regression, the Health Belief Model (HBM), the Theory of Planned Behaviour (TPB), and the Social Ecological Model (SEM), have been widely applied in the study of student health behaviours. However, these models exhibit significant limitations in addressing the complexity of health behaviours and their multidimensional interactions. Linear regression methods typically only identify the independent effects of individual factors and fail to capture nonlinear interactions or causal hierarchies [10]. Theoretical models such as HBM, TPB, and SEM tend to isolate single determinants of health behaviours, neglecting the dynamic interactions between these factors and failing to effectively reflect the interdependencies among behaviours like physical activity, diet, and mental health. Additionally, the linear structure of these models does not fully account for feedback loops in real-world scenarios, which limits their ability to comprehensively explain the formation and changes in health behaviours [11].

In public health, the DEMATEL (Decision-Making Trial and Evaluation Laboratory) method has been widely used to analyse multilevel causal relationships within health systems. For example, Liao and Bercea employed the DEMATEL method to construct a causal relationship model for health promotion, identifying key success factors such as “leadership,” “communication channels,” and “budget,” and proposed corresponding improvement strategies [12]. Similarly, Wang used the DEMATEL-ISM-MICMAC hybrid method to study the influencing factors of public health emergency response mechanisms, revealing the crucial role of social and environmental factors in public health crises [13]. Furthermore, Farhadi used fuzzy DEMATEL and ANP methods to prioritise the factors influencing health service quality in Iran, analysing the interactions among multiple factors and proposing strategies to improve public health service quality [14]. These studies highlight the potential of the DEMATEL method in uncovering multilevel, complex causal relationships within health behaviour systems and provide methodological support for the current study.

In summary, current research primarily focuses on interventions targeting single health behaviours, overlooking the interrelationships between behaviours and the underlying institutional and environmental factors, leading to fragmented intervention outcomes. Though widely applied, traditional models, such as linear regression, the Health Belief Model (HBM), and the Social Ecological Model (SEM), have significant limitations in capturing nonlinear interactions and cross-level causal relationships in health behaviours. Furthermore, the issue of college students’ health behaviours is not merely an isolated phenomenon but a pervasive public health challenge that requires in-depth analysis and intervention from a systemic public health perspective.

Therefore, this study comprehensively analyses the key factors influencing college students’ health behaviours from a multidimensional perspective and offers specific practical recommendations from a public health standpoint. Multiple factors, including psychological, social, and environmental elements, influence the formation and change of college students’ health behaviours, which interact through complex feedback mechanisms to shape health behaviour patterns. However, traditional analytical methods are inadequate in addressing these cross-level interactions and nonlinear feedback mechanisms, thus failing to fully reveal the dynamic changes in health behaviours.

To address this challenge, an integrated analytical framework, Fuzzy-DEMATEL-AISM, is proposed in this study. This framework aims to overcome the shortcomings of existing methods by more effectively capturing the complex interactions and nonlinear relationships within health behaviours, providing both theoretical support and practical guidance for public health interventions. In this framework, the fuzzy-DEMATEL method quantifies the intensity of causal relationships based on expert judgment, while AISM reveals the hierarchical structure among influencing factors. Existing studies typically apply these two methods independently—either for causal inference or structural decomposition—making it challenging to simultaneously handle both causal intensity and hierarchical complexity. To fill this gap, this study is the first to combine the two methods in the health behaviour system, using the outputs from fuzzy-DEMATEL to guide the structural modelling process of AISM. Through this integrated approach, the reliability of causal reasoning and the resolution of hierarchical structures are enhanced under conditions of uncertainty, enabling a more comprehensive understanding of the complexity of health behaviours.

## 2. Research methodology

**Fig 1:**
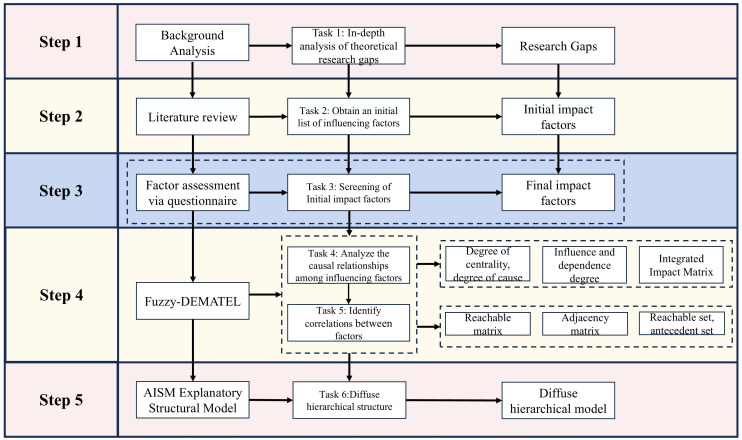
Methodological framework for identifying and analyzing key factors influencing college students’ health behaviors.

This study adopts a five-step framework to identify and analyze key factors influencing college students’ health behaviors: (1) Extensive literature review; (2) factor extraction via expert input; (3) statistical validation using a structured questionnaire; (4) causal analysis using Fuzzy-DEMATEL; and (5) hierarchical modeling with Adversarial Interpretive Structural Modeling (AISM) (see Fig 1). The following sections describe the key methods used in each step.

### 2.1. Literature review and factor identification

To support the systematic identification of influencing factors for model development, a targeted literature review was conducted as an exploratory step, rather than the study’s primary focus. Based on the Web of Science Core Collection and following PRISMA guidelines, 243 articles were initially screened, resulting in 26 empirical studies. Guided by the Social Ecological Model (SEM), 20 key influencing factors were identified and categorized into four dimensions: individual, social/environmental, behavioral, and educational/policy. These factors provided the theoretical basis for the subsequent modeling process (see Table 1).

**Table 1:** Key influencing factors of college students’ health behaviors.

Dimension	Influencing Factors	Reference	Definition
Personal Factors	Health Attitude F1	GA Fava [15] S Park [16]	Individual cognitive and value orientation toward health behaviors; measured using items from the HPLP-II scale
	Age F2	T Kokkinaki [17] CM Vicario [18]	Health behavior characteristics and execution may vary across different age groups of students
	Health Knowledge F3	O Ntshebe [19]	Awareness of nutrition, exercise, and mental health; based on the eHEALS scale
	Mental Health Status F4	JK Podiya [20] WH Kim [21]	Assessed using the General Health Questionnaire (GHQ-12) for stress, anxiety, and emotional stability
	Peer Norms F5	M Nirmala [22]	Perceived behavioral norms among peers regarding health-related actions
	Health Motivation F6	MA Allicock [23]	Internal drive to adopt healthier lifestyles; adapted from motivation self-assessment tools.
Social and Environmental Factors	Social Support F7	Matthews [24] Liang [25]	Emotional and informational support from classmates, friends, and teachers
	Family Economic Status F8	BB Bwalya [26]	The impact of family income on students’ access to health resources
	Family Support F9	MA Allicock [23] K Sai Sushma [27]	Emotional, financial, and behavioral support provided by family members.
	Accessibility of Facilities F10	MS Albadrani [28]	Ease of access to physical activity and recreational spaces
	Sleep Quality F11	Liang [25]	Measured using the Pittsburgh Sleep Quality Index (PSQI).
	Healthy Diet Frequency F12	S Bostan [29] Halloway [30]	Intake frequency of fruits, vegetables, and whole grains; aligned with Chinese Dietary Guidelines
	Academic Stress F13	PA Matson [31] A Mornell [32]	Self-reported stress or fatigue resulting from academic pressure.
Behavioral and Habitual Factors	Physical Activity Frequency F14	L Kakinami [33]	Weekly frequency of moderate to vigorous physical exercise
	Dietary Balance F15	S Bostan [29]	Overall dietary structure including intake of staples, oils, and sugars
	Sedentary Time F16	MA Guerriero [34] AG Price [35]	Average daily duration of uninterrupted sedentary behavior
	Environmental Safety F17	Leung C [36]	Perceived safety of outdoor exercise environments (e.g., lighting, security, noise)
Educational and Policy Support	Participation in Health Education F18	J Jonker [37]	Frequency of attending health lectures, physical check-ups, or online health learning
	Campus Policy Support F19	MG Block Ngaybe [38]	Availability of institutional health promotion policies
	Mental Health Service Accessibility F20	PNY Kwafoa [39] J Fahed-Sreih [40]	Access to school-based psychological counseling resources

### 2.2. Questionnaire design and administration

To empirically evaluate the relevance and reliability of the identified factors, a structured questionnaire was designed using validated scales and expert consultation. After a pilot test with 30 students, the final version was distributed online to 150 students from four universities in Hunan Province, China, via convenience sampling. The sample reflected demographic diversity and contextual relevance (Table 2).

**Table 2:** Basic demographic information table.

Category	Option	Number	Ratio
Gender	Male	70	46.7%
	Female	80	53.3%
Age	18-22	80	53.3%
	23-26	60	40.0%
	27 and above	10	6.7%
Grade	Freshman	30	20.0%
	Sophomore	25	16.7%
	Junior	20	13.3%
	Senior	15	10.0%
	First-year Graduate Student	30	20.0%
	Second-year Graduate Student and above	30	20.0%
Educational Level Professional Background	Bachelor’s Degree	90	60.0%
	Master’s Degree	50	33.3%
	Doctorate and above	10	6.7%
Professional Background	Medical-related Fields	30	20.0%
	Engineering and Technology Fields	40	26.7%
	Social Sciences/Psychology/Education-related Fields	50	33.3%
	Other	30	20.0%

The questionnaire consisted of three sections: (1) demographic information; (2) ratings of potential influencing factors on a five-point Likert scale (1 = no influence, 5 = very strong influence); and (3) open-ended feedback regarding item clarity.

Reliability and construct validity were systematically evaluated through statistical analyses to ensure the internal consistency and theoretical soundness of the measurement instrument. The experts whose profiles are listed in Table 3 were not involved in the questionnaire design but were later consulted in the subsequent modeling phase of the study.

**Table 3:** Expert information.

Category	Options	Number of Experts	Percentage
Nature of the Organization	Public Health Research Institutions	3	30%
	Universities and Educational Institutions	4	40%
	Psychological Health and Education Consulting Institutions	2	20%
	Health Education and Intervention Units	1	10%
Working Experience	5–15 years	5	50%
	More than 15 years	5	50%
Academic Background	Master’s Degree	4	40%
	Doctoral Degree or Higher	6	60%
Title	Associate Senior Title	3	30%
	Senior Title	7	70%

IBM SPSS Statistics (Version 27) was used to test reliability and validity. Internal consistency was confirmed by Cronbach’s α = 0.889. Items with corrected item-total correlation (CITC) < 0.3—specifically “age” and “family economic status”—were excluded. Construct validity was supported by a high KMO value (0.903) and significant Bartlett’s test (p < 0.001).

Descriptive statistics were used to filter items. One-sample t-tests confirmed mean ratings > 3, while standard deviation (SD < 1) ensured consensus. Final variables retained for modeling demonstrated strong psychometric properties (Table 4).

**Table 4:** T-test and ANOVA test.

Items	n	Min	Max	Mean	Std. Deviation	t	p
influencing factors—Gender	152	1.000	5.000	3.559	0.954	7.226	0.000**
Health Knowledge Level	152	1.000	5.000	3.566	0.954	7.314	0.000**
Psychological Health Status	152	1.000	5.000	3.546	0.982	6.854	0.000**
Self-Efficacy	152	1.000	5.000	3.599	0.951	7.763	0.000**
Health Motivation	152	1.000	5.000	3.704	0.969	8.960	0.000**
Sleep Patterns	152	1.000	5.000	3.520	0.976	6.563	0.000**
Family Support	152	1.000	5.000	3.500	0.983	6.269	0.000**
Availability of Campus Facilities	152	1.000	5.000	3.664	0.906	9.044	0.000**
Healthy Eating Environment	152	1.000	5.000	3.704	0.812	10.682	0.000**
Peer Influence	152	2.000	5.000	3.704	0.868	10.003	0.000**
Academic Pressure	152	1.000	5.000	3.757	0.899	10.378	0.000**
Frequency of Physical Activity	152	1.000	5.000	3.750	0.901	10.266	0.000**
Dietary Structure	152	1.000	5.000	3.507	0.956	6.533	0.000**
Sedentary Habits	152	1.000	5.000	3.704	0.975	8.897	0.000**
Screen Time	152	2.000	5.000	3.757	0.906	10.294	0.000**
Participation in Health Education	152	1.000	5.000	3.697	0.921	9.335	0.000**
Support for Health Policies	152	1.000	5.000	3.645	0.902	8.814	0.000**
Psychological Counseling Support	152	1.000	5.000	3.638	0.939	8.380	0.000**

* *p* < 0.05; ** *p* < 0.01

The validated and rigorously screened questionnaire items provided a robust empirical foundation for subsequent analytical modelling. Specifically, these empirically validated factors were directly utilised as quantitative inputs for the Fuzzy-DEMATEL analysis, accurately capturing participants’ perceptions regarding factor importance. To further account for the inherent uncertainty and subjective judgments in assessing complex causal relationships, expert consultations were incorporated exclusively within the Fuzzy-DEMATEL process, complementing the empirical data. This integrated approach ensured enhanced precision and reliability for the causal quantification, thereby providing a reliable basis for the hierarchical structuring conducted in the subsequent AISM analysis.

### 2.3. Causal analysis and hierarchical modeling

#### 2.3.1. Introduction to the integrated method.

To overcome the limitations of traditional structural-analysis methods, this study proposes a hybrid framework combining Fuzzy-DEMATEL and Adversarial Interpretive Structural Modelling (AISM) to simultaneously capture causal intensity and hierarchical topology within the multi-level mechanisms influencing college students’ health behaviours.

Traditional approaches face explicit constraints. Interpretive Structural Modelling (ISM) allows visualisation of hierarchical relationships but relies on binary, presence–absence links that neither quantify causal strength nor reflect nonlinear feedback mechanisms within complex systems [41]. Matrix Impact Cross-Reference Multiplication (MICMAC) emphasises the identification of key driving variables through cross-impact matrices, yet it lacks hierarchical granularity and dynamic adaptability [42]. While suitable for modelling interdependencies, the Analytic Network Process (ANP) offers limited capacity for identifying vertical stratification or quantifying directional causal weights in layered systems [43].

Compared to these models, the proposed Fuzzy-DEMATEL-AISM framework provides a more refined and integrated solution. First, Fuzzy-DEMATEL enables the quantification of weighted causal intensities under expert uncertainty. These outputs are then used to generate a total influence matrix, which serves as the input for AISM. Second, AISM applies an adversarial layering algorithm to this matrix to construct a transparent and interpretable hierarchical structure, where upper and lower levels are systematically delineated.

**Fig 2:**
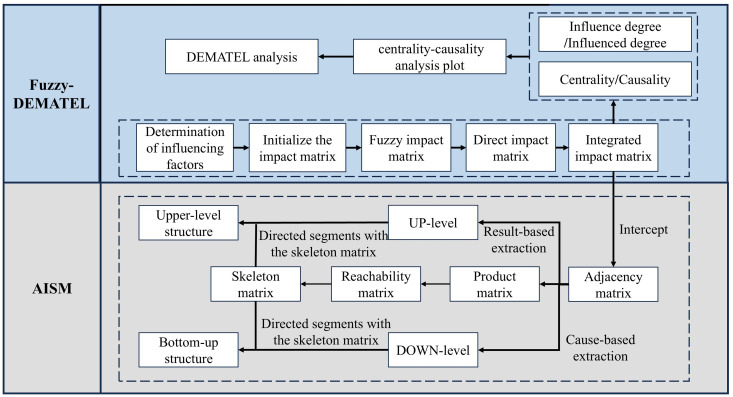
Integrated framework for analyzing causal relationships and hierarchical structure of factors influencing college students’ health behaviors.

Fig 2 illustrates that this integration allows one workflow for threshold-based skeleton reduction and structural decomposition. By capturing strength and structure simultaneously, the framework significantly outperforms conventional models in disentangling causal complexity, especially under conditions of uncertainty and feedback. This methodological advancement offers a powerful analytical tool for understanding health-behaviour systems and lays a rigorous empirical foundation for formulating effective public-health intervention strategies in higher education.

#### 2.3.2. Construction of influencing factors indicators.

Based on a comprehensive literature review, 18 key factors influencing college students’ health behaviors were identified. These factors were classified into four dimensions: individual psychological cognition, social and environmental support, behavioral habits, and educational and policy support. Each factor (F₁–F₁₈) was drawn from validated measurement tools or authoritative guidelines. All items were rated on a five-point Likert scale (1 = not important at all, 5 = very important) to evaluate perceived importance. Detailed definitions of each factor are provided in Table 5.

**Table 5:** Key influencing factors and dimensions.

Dimension	Name of influencing factor	Definition
Personal Factors	F1 Health Attitude	Individual cognitive and value orientation toward health behaviors; measured using items from the HPLP-II scale
	F2 Health Knowledge	Awareness of nutrition, exercise, and mental health; based on the eHEALS scale.
	F3 Mental Health Status	Assessed using the General Health Questionnaire (GHQ-12) for stress, anxiety, and emotional stability.
	F4 Peer Norms	Perceived behavioral norms among peers regarding health-related actions.
	F5 Health Motivation	Internal drive to adopt healthier lifestyles; adapted from motivation self-assessment tools.
Social and Environmental Factors	F6 Social Support	Emotional and informational support from classmates, friends, and teachers.
	F7 Family Support	Emotional, financial, and behavioral support provided by family members.
	F8 Accessibility of Facilities	Ease of access to physical activity and recreational spaces.
	F9 Sleep Quality	Measured using the Pittsburgh Sleep Quality Index (PSQI).
	F10 Healthy Diet Frequency	Intake frequency of fruits, vegetables, and whole grains; aligned with Chinese Dietary Guidelines.
	F11 Academic Stress	Self-reported stress or fatigue resulting from academic pressure.
Behavioral and Habitual Factors	F12 Physical Activity Frequency	Weekly frequency of moderate to vigorous physical exercise.
	F13 Dietary Balance	Overall dietary structure including intake of staples, oils, and sugars.
	F14 Sedentary Time	Average daily duration of uninterrupted sedentary behavior.
	F15 Environmental Safety	Perceived safety of outdoor exercise environments (e.g., lighting, security, noise).
Educational and Policy Support	F16 Participation in Health Education	Frequency of attending health lectures, physical check-ups, or online health learning.
	F17 Campus Policy Support	Availability of institutional health promotion policies.
	F18 Mental Health Service Accessibility	Access to school-based psychological counseling resources.

#### 2.3.3. Fuzzy-DEMATEL procedure.

To identify causal relationships among influencing factors, the Fuzzy-DEMATEL method was applied. Ten experts, whose professional backgrounds are summarized in Table 3, evaluated the direct influence between each factor pair using a 0–4 scale (0 = no influence, 4 = strong influence). These scores were converted into triangular fuzzy numbers to account for subjective uncertainty, using linguistic labels such as “slight,” “moderate,” and “significant.” The resulting fuzzy direct-influence matrix was then defuzzified using the CFCS (Converting Fuzzy Data to Crisp Scores) method, producing a crisp direct influence matrix.

**Fig 3:**
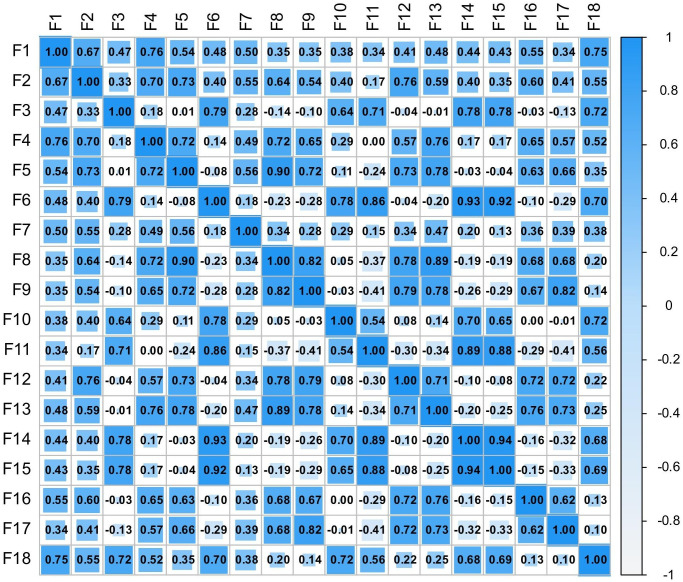
Aggregate impact matrix C.

Following normalization and aggregation procedures, a total influence matrix was constructed, integrating both direct and indirect effects among factors. This matrix, presented as Fig 3, reveals the comprehensive impact pathways within the system and serves as a foundational input for the subsequent calculation of system metrics.

Based on the total influence matrix, four key indicators were computed: influence degree, degree of being influenced, centrality, and causality. These metrics allowed the classification of each factor as either a cause or an effect. The results were visualized in a causal relationship diagram, with full computational procedures detailed in Appendix A.

#### 2.3.4. AISM-based hierarchical structuring process.

In the second stage, Adversarial Interpretive Structural Modeling (AISM), adapted from Li et al. [44], was employed to construct a multilevel structure of influencing factors. Unlike traditional ISM, AISM integrates two opposing extraction rules—result-priority and cause-priority—to compare hierarchical outputs and identify structural asymmetries, revealing dominant, transitional, and foundational elements.

The total influence matrix from Fuzzy-DEMATEL was converted into a binary adjacency matrix using a threshold (λ = mean + standard deviation) [45], with values ≥ λ coded as 1 (significant influence) and others as 0. A reachability matrix was then generated through Boolean operations, followed by node and edge reduction to form a generalized skeleton matrix.

Hierarchy levels were extracted via set intersection: factors satisfying the UP-type rule were placed at higher levels, while those matching the DOWN-type rule were placed lower. AISM also identifies “activity elements,” which show level differences between the two hierarchies, reflecting dynamic and context-dependent roles. The resulting hierarchy maps the system’s underlying causal topology. Notably, the term “adversarial” refers to the structural logic, not to machine learning. Full computational procedures are detailed in Appendix A.

#### 2.3.5. Integration and implementation of methods.

To integrate causal identification with structural modeling, this study sequentially applied Fuzzy-DEMATEL and AISM. Fuzzy-DEMATEL quantified the causal strengths among factors and generated a total impact matrix, which served as the structural input for AISM. AISM then transformed this matrix into a binary adjacency matrix using a threshold, enabling hierarchical extraction through topological analysis.

This integrated approach forms a closed-loop framework that links quantitative evaluation with multilevel system interpretation, enhancing the robustness of complex variable analysis. All computations were performed in Python 3.12 using standard libraries (NumPy, pandas, SciPy, NetworkX) and custom scripts for defuzzification and matrix transformation. Appendix A, Table 6 provides a detailed summary of all key model parameters used in the Fuzzy-DEMATEL and AISM process, along with brief explanations of their theoretical or practical relevance.

**Table 6:** Model Parameters and Rationale for Fuzzy-DEMATEL and AISM Implementation.

Parameter/ Procedure	Model Component	Assigned Value/ Rule	Rationale
Expert influence rating scale	Fuzzy-DEMATEL	0 (no influence) to 4 (very strong influence)	Standardized scale in DEMATEL applications to assess directional influence strength.
Fuzzification method	Fuzzy-DEMATEL	Triangular fuzzy numbers with linguistic terms (“slight”, “moderate”, “significant”)	Captures uncertainty in expert judgment with intuitive interpretation.
Defuzzification approach	Fuzzy-DEMATEL	CFCS (Converting Fuzzy Data to Crisp Scores)	Balances computational simplicity and accuracy; suitable for fuzzy matrix processing.
Threshold for adjacency matrix (λ)	AISM	λ = mean + standard deviation of total impact matrix	Filters significant influences while minimizing noise for binary matrix conversion.
Hierarchical extraction rules	AISM	UP-type and DOWN-type rule sets	Enables bidirectional analysis of structural hierarchy, reflecting cause–effect asymmetry.
Structural simplification method	AISM	Boolean matrix iteration and node/edge pruning	Enhances clarity by eliminating redundant paths and focusing on core structure.
Programming environment	Implementation platform	Python 3.12	Open-source and widely adopted platform for computational modeling.
Key libraries used	Implementation platform	NumPy, pandas, SciPy, NetworkX	Provides robust support for numerical computation and graph-based analysis.

### 2.4. Ethical considerations

This study received ethical review and approval from the Institutional Review Board of Changsha University of Science and Technology (Approval No. CSUST-IRB-2025-042) prior to data collection. Participants were prospectively recruited from January 10 to February 5, 2025 through online dissemination of the questionnaire. Before beginning the survey, each participant was presented with a written informed consent form outlining the study objectives, procedures, voluntary participation, data confidentiality, and the right to withdraw at any point. Informed consent was obtained electronically by checking the “Yes” box and digitally signing via the Wenjuanxing platform prior to accessing the questionnaire. All participants were aged 18 years or older, and therefore, parental or guardian consent was not required.

## 3. Results

### 3.1. Distribution and ranking of factors in influence, influenced, causality, and centrality

Table 7 presents the ranking results for four key metrics—Influence, Influenced, Causality, and Centrality. Factor F3 exhibits the highest influence, with a value of 3.257, ranking 1st, followed by F2 (3.084, ranked second). The factors with the lowest influence are F1 (0.668) and F14 (0.952), ranked 18th and 17th, respectively. Regarding being influenced, F5 has the highest influenced value (3.033), ranked 1st, F1 has the lowest influenced value (0.349), ranked 18th, and F16’s influenced value is 2.595, ranked third. Regarding causality, F2 has the highest (1.181), ranked 1st, with F3’s causality value at 0.658, ranked fifth. The factors with the lowest causality values include F12 (−1.320, ranked 18th), F13 (−0.945, ranked 15th), and F14 (−1.121, ranked 16th). For centrality, F3 leads with a value of 5.857, ranked 1st, followed by F5 with a centrality value of 5.400, ranked third. The factors with the lowest centrality are F1, F2, and F14, ranked 18th, 6th, and 17th, respectively.

**Table 7:** Degrees of influence, influenced, causality, and center.

Factor	Influence	Rank	Influenced	Rank	Causality	Rank	Center	Rank
**F1**	0.668	18	0.349	18	0.320	9	1.017	18
**F2**	3.084	2	1.904	14	1.181	1	4.988	6
**F3**	3.257	1	2.600	2	0.658	5	5.857	1
**F4**	2.704	7	2.536	4	0.168	10	5.239	4
**F5**	2.366	8	3.033	1	−0.667	13	5.400	3
**F6**	1.479	14	2.335	7	−0.856	14	3.814	12
**F7**	2.891	4	2.021	13	0.870	3	4.912	7
**F8**	2.101	10	1.666	17	0.435	7	3.766	14
**F9**	1.926	12	2.092	11	−0.166	12	4.017	10
**F10**	2.082	11	1.706	16	0.376	8	3.788	13
**F11**	2.288	9	2.187	9	0.101	11	4.475	9
**F12**	1.200	15	2.520	5	−1.320	18	3.721	15
**F13**	1.485	13	2.430	6	−0.945	15	3.914	11
**F14**	0.952	17	2.073	12	−1.121	16	3.025	17
**F15**	1.085	16	2.209	8	−1.124	17	3.294	16
**F16**	3.034	3	2.595	3	0.439	6	5.629	2
**F17**	2.881	5	2.150	10	0.731	4	5.031	5
**F18**	2.783	6	1.862	15	0.921	2	4.645	8

**Fig 4:**
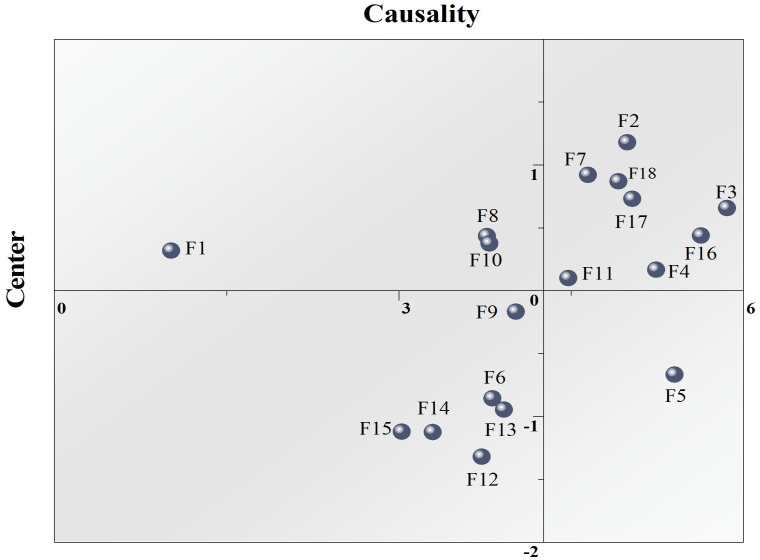
Cause-effect distribution and centrality structure of key factors influencing college students’ health behaviors.

Fig 4 illustrates the relationship between causality and centrality, reflecting the distribution pattern of factors across these two dimensions. The factors located in the upper-right quadrant, such as F3 (causality 0.658, centrality 5.857), F17 (causality 0.731, centrality 5.031), and F18 (causality 0.921, centrality 4.645), exhibit high causality and centrality, indicating their significant role in both causal influence and network centrality. Factors F2 (causality 1.181, centrality 4.988) and F4 (causality 0.168, centrality 5.239) are positioned in the lower-right quadrant, showing high causality but slightly lower centrality, suggesting their importance in causal relationships while being less central in the network structure. In the lower-left quadrant, factors F12 (causality −1.320, centrality 3.721), F13 (causality −0.945, centrality 3.914), and F14 (causality −1.121, centrality 3.025) display weak causality and lower centrality, indicating their limited influence and connectivity in the network. F1 (causality 0.320, centrality 1.017) is located in the upper-left quadrant, reflecting its lack of significant causal impact and central position within the network.

Overall, the distribution in Fig 4 aligns with the data in Table 7, where factors with high causality and centrality are generally located in the upper-right quadrant. In contrast, those with low causality and centrality are positioned in the lower-left quadrant.

### 3.2. Hierarchical Influence Structure Based on AISM

**Fig 5:**
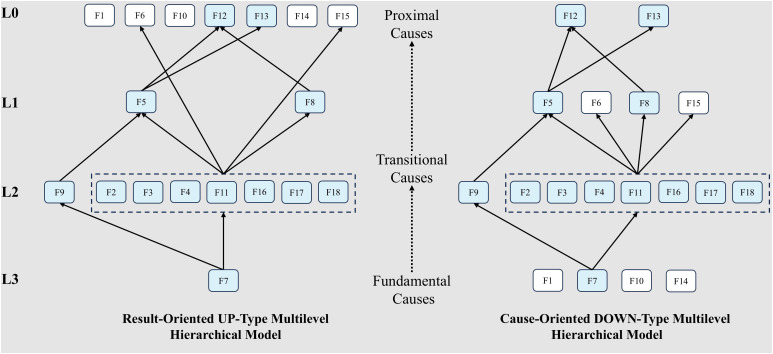
Multilevel hierarchical structure of factors influencing college students’ health behaviors derived from AISM.

The AISM analysis reveals the hierarchical structure of the factors, as shown in Fig 5. Family support (F7) is positioned at the lowest level in this structure. Although other factors do not influence F7, they influence other factors through multiple outward connections.

Next, in the middle layer, health knowledge (F2), mental health status (F3), and health motivation (F5) are influenced by upstream factors and transmit these effects to behavioural outcomes.

Further, the factors at the proximal layer, such as physical activity frequency (F12) and dietary balance (F13), are at a lower level in the structure. While the middle layer factors influence these factors, they do not transmit influence further, which aligns with their lower causality scores.

Finally, the factors at the outer layer, including peer influence (F9), sleep quality (F6), and accessibility of exercise spaces (F15), show weaker connectivity. These factors have fewer interrelationships with other factors.

## 4. Discussion

### 4.1. Key findings

Based on the empirical evidence presented in the preceding Results section, a comprehensive analysis indicates that three interconnected theoretical contributions have been made to health behaviour. First, family support (F7) has been confirmed to reside at the foundational level of the hierarchical model, acting as an initiating driving force whose influence is continuously transmitted to downstream psychological and motivational factors. Second, the “knowledge–motivation–psychology” mediation chain has been elucidated, whereby mental health status (F3), health motivation (F5), and health knowledge (F2) jointly constitute the cognitive–affective core; the pathway F7 → F3/F2 → F5 → F12/F13 demonstrates that motivation is the pivotal hub through which affective support and cognitive resources are transformed into sustained health behaviors [45]. Finally, the findings have revealed that college students’ health behaviours are dynamically co-shaped by familial, institutional, and psychological factors, highlighting the necessity of adopting a multi-level perspective to explain and intervene in this population’s health practices.

### 4.2. Explanation and mechanistic analysis

Family support (F7) has been identified as a foundational driving force for college students’ mental health and behavioural motivation by providing emotional care and a sense of security. In line with the family‐stress model and social-support theory, adequate family backing can be leveraged as a self-regulatory resource that buffers stress and fosters positive emotions, enhancing mental-health status and stabilising behavioural expectations [46]. In collectivistic cultures, family support is particularly significant, as it often transcends individual psychological independence and continues to play a crucial role in developing motivation and coping strategies during early adulthood [47]. Parental understanding, encouragement, and recognition have been shown not only to reduce depression and anxiety but also to strengthen self-efficacy and self-worth, thus increasing students’ confidence and willingness to pursue health goals [48]. Empirical studies have further demonstrated that college students receiving high levels of family support exhibit stronger intrinsic motivation in academic and health domains, such as more active engagement in physical exercise and adopting health-management measures [49]. Accordingly, the present model positions family support as the root driver of multiple psychological and motivational variables, with its influence permeating every stage of health-behaviour formation.

Health knowledge (F2) is regarded as the cognitive substrate of health behaviour and has markedly enhanced health motivation (F5). When individuals clearly understand the risks and benefits of a given behaviour, stronger intrinsic motivation to change is likely to emerge; conversely, knowledge deficits often result in inadequate motivation and impede the correction of unhealthy habits [50]. This pattern accords with the knowledge–attitude–practice(KAP) framework, wherein knowledge affects attitudes—an expression of motivation—thereby indirectly governing behaviour [51]. As health motivation intensifies, college students become more inclined to engage in physical exercise and maintain a balanced diet, improving mental health status (F3). Elevated motivation is also associated with lower disease risk and higher subjective well-being [52]. In contrast, persistent stress and negative emotions have been found to erode motivation and undermine the maintenance of health behaviours, creating a detrimental cycle [53]. Hence, a positive feedback loop—“knowledge → motivation → behaviour → psychology”—is proposed, in which health knowledge fosters motivation, motivation drives behaviour, behaviour enhances psychological well-being, and improved psychological states subsequently reinforce motivation and behaviour [54].

Finally, cultivating health behaviours results from dynamic interactions among family environments, institutional supports, and individual psychological factors. The social-ecological model posits that multilevel contexts—including family, peers, school systems, and policies—and personal attributes shape individual behaviour [55]. Externally, a supportive family climate—characterised by parental companionship, health education, encouragement, and higher socioeconomic status—has substantially increased college students’ willingness and frequency to participate in physical activities [56]. At the same time, comprehensive campus policies and facilities provide favourable conditions for practising health behaviours [57]. Internally, mental health, health motivation, and self-efficacy determine whether external support can be translated into sustained behaviours. Evidence indicates that students in good psychological condition participate more actively in exercise and maintain higher dietary quality. In contrast, excessive stress or negative emotions suppress exercise motivation and deteriorate diet quality [58]. Consequently, family and school supports exert synergistic effects by shaping individual psychology and motivation: external resources and opportunities interact with internal drives, complementing one another and jointly promoting—or impeding—the maintenance and enhancement of a healthy lifestyle among college students [59].

### 4.3. Comparison with previous research

Family support (F7) was identified as the foundational driver within the hierarchical model. This finding accords with numerous investigations adopting socio-ecological or family-stress perspectives, where parental care was likewise reported to buffer stress and enhance self-efficacy during the college years [60,61]. In contrast, many cross-sectional surveys based on North American samples documented a rapid attenuation of family influence after students left home, with peer influence becoming predominant [62]. The opposite pattern observed here can be partly attributed to AISM’s capacity to capture indirect pathways, thereby revealing the deep penetration of familial factors into subsequent psychological–motivational chains; additionally, the respondents were drawn from a collectivistic culture in which emotional and financial dependence on parents remains pronounced [63]. This divergence indicates that cultural and methodological constraints limit the ‘waning family effect’ hypothesis. Specifically, family influence tends to remain strong throughout young adulthood in collectivistic cultures, contrasting with the rapid attenuation observed in individualistic cultures. This suggests that the ‘waning family effect’ hypothesis may not fully capture the dynamics in non-Western cultural contexts, where emotional and financial dependence on parents remains more pronounced even in university years [64].

The ‘knowledge–motivation–psychology’ chain (F7 → F3/F2 → F5 → F12/F13) was validated in line with the KAP framework and the HAPA model, both of which posit that knowledge must be channelled through motivation before it can translate into action. However, the present study expands these models by positioning mental health (F3) and health knowledge (F2) as parallel antecedents of motivation, offering a nuanced perspective on the origins of the ‘knowledge–action gap.’ This extension provides new insights into how psychological well-being serves not just as an outcome, but also as a catalyst that enables knowledge to convert into action [65,66]. The present study extends these theories by positioning mental health (F3) and health knowledge (F2) as parallel antecedents of motivation, thereby refining the origins of the “knowledge–action gap”. Many intervention trials have treated mental health as merely an outcome variable, often overlooking its role as a mediator in the knowledge-behaviour relationship [67]. This gap has left the persistence of weak behavioural change despite improved knowledge unexplained. Our study addresses this by positioning mental health as a critical mediator, illustrating how psychological well-being can enhance the effectiveness of health knowledge in driving sustainable behaviour change [68]; the current findings demonstrate that a positive psychological state is fertile ground for knowledge germinating into action. Although a few studies did not detect a significant impact of knowledge on motivation [69], such discrepancies may stem from the more refined latent-variable measurement employed here, which increased statistical power and the wider variance in health knowledge within the present sample.

By integrating familial, institutional, and psychological factors into a single hierarchical model, this study not only corroborated the socio-ecological premise that behaviour is generated through multi-level interactions [70] but also quantified the sequential positions of the constituent factors, revealing that campus facilities and policies operate chiefly by strengthening health motivation. This result provides a prioritisation framework for resource allocation in higher education and enriches recent systematic reviews of “multi-level” interventions with empirical detail. Furthermore, while several studies have placed peer influence at the core of their causal models [71], our findings assign it a relatively low causal weight. This may be attributed to the strong influence of familial and institutional forces in collectivistic contexts, which dilute peer effects. Additionally, AISM’s ability to filter out weak links to psychological variables may further explain the limited role of peer influence in the present model.

The results suggest that the influence of certain variables, such as peer influence, mental health, and health knowledge, may be context-specific. For instance, in some educational environments, the impact of family and institutional factors may be more significant than peer influence, especially when family involvement is high [72]. Similarly, these variables may have different effects across subgroups, such as students from different cultural backgrounds or those with varying levels of resource access, who may respond differently to mental health and health knowledge. Therefore, the impact of mental health and health knowledge on behaviour may vary according to factors such as socioeconomic status, age, or gender, leading to differences in the outcomes of health behaviour formation across different groups [73,74].

### 4.4. Practical implications: Multilevel intervention framework

**Fig 6:**
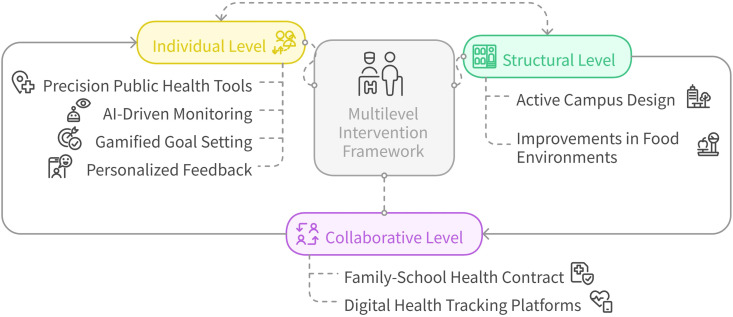
Structure–collaboration–individual framework for enhancing health behaviors in university educational settings.

This study proposes a multilevel intervention framework—Structure–Collaboration–Individual—to address student health behaviors in university settings (see Fig 6). At the structural level, interventions such as active campus design (e.g., walking paths, cycling routes, standing desks) can reduce sedentary behavior by approximately 19% [75], while improvements to food environments promote healthier dietary habits as part of broader educational design. At the collaborative level, a Family–School Health Contract supported by digital health-tracking platforms helps clarify shared responsibilities between educators and families, improving physical activity adherence by up to 41% (2). This aligns with the WHO-endorsed model for strengthening school–family health alliances. At the individual level, precision public health tools—such as AI-driven monitoring, gamified goal setting, and personalized feedback—enable students to translate school-based health literacy into sustainable behavior patterns. Collectively, this framework reflects the integration of policy, environment, and educational action into a closed-loop system for health behavior change in formal education settings [76].

### 4.5. Theoretical significance

The key findings of the present study advance health-behaviour theory in three substantive respects. First, by confirming that family support continues to serve as a proximal driver during the university stage, the conventional socio-ecological assumption that familial influence diminishes steadily with age is broadened, and the family is recast as a sustained regulator spanning adolescence and early adulthood.

Second, a parallel dual-mediator chain of “health knowledge–mental health–health motivation” has been proposed and validated. In this chain, a positive psychological state is identified as a behavioural outcome and a prerequisite for the internalisation of knowledge into motivation, thereby refining the KAP and HAPA accounts of the “knowledge–action gap.”

Third, by quantifying the cascading sequence of familial, institutional, and psychological factors, our hierarchical model assigns explicit path weights within the socio-ecological architecture and shows that macro-level variables act primarily by activating individual motivation.

Building on this systems perspective, we demonstrate how the integrated Fuzzy-DEMATEL-AISM framework overcomes the structural blind spots of classic individual-level theories such as the Health Belief Model (HBM) and the Theory of Planned Behaviour (TPB). Whereas HBM and TPB foreground cognitive appraisals—perceived threat, attitudes, and intentions—while treating contextual constraints as exogenous noise [77], our two-stage procedure first quantifies the strength of structural and psychosocial links with Fuzzy-DEMATEL and then embeds these weighted links into AISM’s hierarchy. The resulting longitudinal map specifies how policies, facilities, and family systems cascade through psychosocial mediators to shape concrete behaviours, thereby restoring the “structural focus” absent from HBM/TPB and revealing leverage points for intervention. This replicable paradigm shows how contextual forces can be integrated into cognition-oriented models without sacrificing analytical precision [78].

These advances collectively refine prevailing health-behaviour theories by embedding quantifiable structural pathways into cognition-oriented frameworks and identifying empirically validated leverage points across the socio-ecological spectrum. This synthesis offers a scalable model that integrates structural constraints, cognitive–affective mechanisms, and feedback dynamics, capturing the complexity of modern health behaviour.

## 5. Conclusion

This study innovatively integrates Fuzzy-DEMATEL with Adversarial Interpretive Structural Modelling (AISM) to develop a systematic analytical framework for identifying and analysing the multi-level causal relationships influencing college students’ health behaviours. The innovation lies in the seamless combination of causal inference and structural modelling, effectively addressing the challenge in existing research where these methods are typically applied independently, making it difficult to handle both causal intensity and hierarchical complexity concurrently. Specifically, Fuzzy-DEMATEL quantifies the strength of causal relationships, while AISM reveals the hierarchical structure among influencing factors. Theoretically, this study extends the traditional socio-ecological model by confirming that family support plays a foundational role in shaping health behaviours among college students, challenging the prevailing notion that familial influence diminishes during late adolescence. Moreover, the study proposes and validates a dual-mediation chain of “health knowledge–mental health–health motivation,” highlighting the critical role of health knowledge and psychological well-being in stimulating health motivation, thereby providing a refined framework to understand the “knowledge–action gap.” Empirically, the findings indicate that college students’ health behaviours emerge from the dynamic interactions of familial, institutional, and psychological factors, underscoring the importance of a multi-level perspective in explaining health behaviours. These insights provide both theoretical support and practical guidance for designing health interventions in higher education, offering a comprehensive framework for addressing health behaviour formation.

The multilevel intervention framework proposed in this study provides systematic guidance for health policy in higher education from a public health perspective. Structurally, optimising campus and food environments reduces sedentary behaviour and promotes healthier eating habits, thereby addressing environmental factors that influence student health. Collaboratively, strengthening school-family partnerships through health contracts and digital platforms enhances physical activity adherence, fostering a supportive network that integrates both institutional and familial resources. At the individual level, precision public health tools, such as personalised health monitoring and feedback systems, effectively support long-term behaviour change, empowering students to maintain healthier lifestyles. Overall, this framework emphasises the integration of policy, environment, and education, offering theoretical insights and practical solutions for university health interventions, while reinforcing the critical role of public health in promoting sustainable health behaviour change within academic settings.

Despite this study’s methodological innovation and analytical rigour, several limitations should be acknowledged. First, the reliance on expert scoring introduced a degree of subjectivity, potentially affecting the objectivity of the results. Second, the sample, primarily drawn from universities in central China, limits the generalizability of the findings across different regions and cultural contexts. Additionally, the cross-sectional design restricts the ability of the model to capture temporal dynamics in behavioural evolution, preventing the establishment of causal relationships over time.

Future research can address these limitations in several ways. Firstly, Longitudinal panel studies with 6–12-month follow-up intervals could track changes in health behaviours and motivational drivers, enabling temporal mapping of causal pathways. Secondly, Cross-national validation studies in collectivistic and individualistic cultures (e.g., China vs. the U.S.) would help examine whether the identified causal structures and motivational chains are culturally robust or context-specific. Furthermore, integrating wearable-sensor data (e.g., activity trackers or sleep monitors) with self-reported motivation scores could support dynamic modelling of health behaviour trajectories. Additionally, future studies should consider adopting participatory co-creation approaches, involving students, families, and universities, to identify context-specific influencing factors and enhance the practical relevance and scalability of intervention strategies.

## Appendix A: Mathematical Derivations of the Fuzzy-DEMATEL and AISM Methods

### A.1. Triangular Fuzzy Numbers and Membership Function

A triangular fuzzy number $\overset{\sim }{N}$ is shown as a triplet (l,m,r). The membership function $\mu_{\overset{\sim }{N}}(x)$ is defined as:


$$\begin{array}{c} \mu_{\tilde{N}}(x)=\left\{ \begin{array}{c} \frac{x-l}{m-l},\;l\leq x\leq m\\ \frac{r-x}{r-m},\;m\leq x\leq r\\ \text{0},\;x<l\;\text{or}\;x>r \end{array}\right.\; \end{array}$$ (1)

### A.2. Defuzzification Using CFCS

The CFCS (Converting Fuzzy Data into Crisp Scores) method is used to transform fuzzy matrices into crisp values. The procedure includes the following steps:

1 Normalization:

$$xl_{ij}^{k}=(l_{ij}^{k}-minl_{ij}^{k})/\Delta_{min}^{max}$$ (2)

$$xm_{ij}^{k}=(m_{ij}^{k}-minl_{ij}^{k})/\Delta_{min}^{max}$$ (3)

$$xr_{ij}^{k}=(r_{ij}^{k}-minl_{ij}^{k})/\Delta_{min}^{max}$$ (4)

Where $\Delta_{min}^{max}=maxr_{ij}^{k}-minl_{ij}^{k}$。

2 The normalized values for the left and right sides are calculated as:

$$xls_{ij}^{k}=xm_{ij}^{k}/(\text{1}+xm_{ij}^{k}-xl_{ij}^{k})$$ (5)

$$xrs_{ij}^{k}=xr_{ij}^{k}/(\text{1}+xr_{ij}^{k}-xm_{ij}^{k})$$ (6)

3 The total normalized value is then calculated as:

$$x_{ij}^{k}=[xls_{ij}^{k}(\text{1}-xls_{ij}^{k})+xrs_{ij}^{k}xrs_{ij}^{k}]/[\text{1}-xls_{ij}^{k}+xrs_{ij}^{k}]$$ (7)

4 Defuzzification for the k−th expert’s evaluation:

$$z_{ij}^{k}=minl_{ij}^{k}+x_{ij}^{k}\Delta_{min}^{max}$$ (8)

5 The evaluations from p experts are combined to get the defuzzified direct impact matrix A:

$$z_{ij}^{k}=minl_{ij}^{k}+x_{ij}^{k}\Delta_{min}^{max}$$ (9)

### A.3. DEMATEL Computation

1 Normalized impact matrix B:

$$B=\frac{x_{ij}}{\text{max}\left(\sum_{j=\text{1}}^{n}\;\;x_{ij}\right)}$$ (10)

2 Total Impact Matrix:

$$T=\left(B+B^{\text{2}}+\cdots +B^{k}\right)=\sum_{k=\text{1}}^{\infty }\;B^{k}=B(I-B{)}^{-\text{1}}$$ (11)

Where I represents the identity matrix.

3 Calculation of Indicators:

Impact Degree:


$$D_{i}=\sum_{j=\text{1}}^{n}\;x_{ij},(i=\text{1},\text{2},\ldots ,n)$$ (12)

Received Influence:


$$C_{i}=\sum_{j=\text{1}}^{n}\;x_{ji},(i=\text{1},\text{2},\ldots ,n)$$ (13)

Centrality:


$$M_{i}=D_{i}+C_{i}$$ (14)

Causality:


$$R_{i}=D_{i}-C_{i}$$ (15)

### A.4. AISM Computation

1 Adjacency Matrix (Thresholding):

$$A_{ij}=\left\{ \begin{array}{cc} C_{ij}, & \text{ if }C_{ij}\geq T\\ \text{0}, & \text{ if }C_{ij}<T \end{array}\right.\;$$ (16)

2 Reachability Matrix:

$$(A+I{)}^{K-\text{1}}\neq (A+I{)}^{K}=(A+I{)}^{K+\text{1}}=M$$ (17)

This step produces the reachability matrix M.

3 Edge Reduction (Skeleton Matrix):

$${\text{S}}^{\prime }={\text{M}}^{\prime }-{\left({\text{M}}^{\prime }-\text{I}\right)}^{\text{2}}-\text{I}$$ (18)

4 Hierarchical Extraction:

$$J\left(e_{i}\right)=R\left(e_{i}\right)\;\text{ UP Topology Hierarchy Extraction}$$ (19)

$$J\left(e_{i}\right)=Q\left(e_{i}\right)DOWN\;\;\text{topology level extraction}$$ (20)

## Supporting information

S1 File Survey data. (XLSX)
